# Workforce Development Programs for Older Adults: Participant, Program, and Policy Insights

**DOI:** 10.1093/geroni/igaf122.124

**Published:** 2025-12-31

**Authors:** Mary Lou Ciolfi, Jennifer Crittenden, Rachel Coleman

**Affiliations:** UMaine Center on Aging, Orono, Maine, United States; University of Maine Center on Aging, Orono, Maine, United States; University of Maine Center on Aging, Orono, Maine, United States

## Abstract

The confluence of increased longevity and critical labor shortages presents opportunities for older adult workforce development (WFD) programming that meets the needs of older adults, employers, and communities. Research on an AmeriCorps Seniors funded demonstration of seven diverse workforce development programs reveals important insights into older worker motivation, program structures and processes, external environments, and local, state, and federal policy that can inform workforce policy and programming into the future. The variety of geographic locations, labor supply, and work sector focus of the seven programs contributes to compelling questions around how to attract older workers to compensated employment, thereby decreasing critical labor shortages in regions and commercial sectors around the country. This presentation highlights early findings from the University of Maine Center on Aging’s research identifying core components of successful WFD programs. Data include survey (N = 191) and focus group and interview data (N = 45) collected across the seven programs. Year One findings illuminate promising ideas related to program design and implementation, messaging, incentives and barriers, government and higher education involvement and support, and the community and demographic data needed to boost program success, worker satisfaction, and partner collaboration. Research findings highlight valuable WFD program outcomes including participant social and emotional wellness and use of a cohort model. Lessons learned include use of specific recruitment messaging and platforms, adapting training to older workers, aligning skills and employment opportunities, and securing community partnerships to create successful, regionally responsive workforce programming that strengthens individuals, organizations, communities, and employment sectors.

